# Hash functions in nucleotide sequence analysis

**DOI:** 10.1101/gr.281453.125

**Published:** 2026-05

**Authors:** Ke Chen, Xiang Li, Qian Shi, Mingfu Shao, Paul Medvedev

**Affiliations:** 1 Department of Computer Science and Engineering, The Pennsylvania State University, University Park, Pennsylvania 16802, USA;; 2 Huck Institutes of the Life Sciences, The Pennsylvania State University, University Park, Pennsylvania 16802, USA;; 3 Department of Biochemistry and Molecular Biology, The Pennsylvania State University, University Park, Pennsylvania 16802, USA

## Abstract

Randomness is a powerful tool in the design and analysis of algorithms and data structures for nucleotide sequence data. Nucleotide sequences are not themselves random but are often randomized using hash functions. Despite their widespread use in genomics, there is no comprehensive review of the types of hash functions used and their various applications. In this survey intended for bioinformatic methods developers, we divide hash functions into four categories: scattering hash functions, permutations, minimum perfect hash functions, and locality-sensitive hash functions. For each category, we provide examples of both general-use hash functions that have been applied in nucleotide sequence analysis and hash functions that have been designed specifically for nucleotide sequence analysis. We highlight their salient properties, commonalities, differences, and application areas.

Randomness is a powerful concept in nucleotide sequence analysis. Many algorithms are known to perform better on data that is random, because random data is unlikely to exhibit the intricate structures that can reduce performance (Shaw and Yu 2023). Unfortunately, DNA sequence data is not random. For example, the set of all *k*-mers (i.e., substrings of length *k*) from a genome differs substantially from a set of random *k*-mers. Among the differences are homopolymers, which are more likely to appear in genomes than by chance. Also, adjacent *k*-mers overlap by *k* − 1 characters, which does not happen frequently in a set of random *k*-mers. The problem of nonrandomness is not unique to nucleotide sequence data and is common across real-world data sets from many domains.

Even though the data is not itself random, it can nevertheless be randomized with the help of hash functions. A hash function associates a pseudorandom hash value with each input element, while simultaneously ensuring that identical items produce the same hash value. Downstream algorithms can then work with the hash value instead of the original input element. Such hashing can improve an algorithm’s accuracy, speed, and/or memory usage. A ubiquitous genomic example of randomization’s power is that the size of a winnowed minimizer sketch is greatly reduced by replacing the deterministic lexicographical *k*-mer order with a randomized one (Marçais et al. 2019b).

The term “hash” originates from the food preparation process of “chop and mix,” which mirrors its function in hashing algorithms that similarly “chop and mix” data to generate hash values (Knuth 1968). The concept of hashing was first proposed by Luhn (1953) and formally named in Morris (1968). Since then, hashing techniques have evolved from simple randomization to sophisticated adaptive methods that consider locality, structure, label information, and data security. These advanced methods have enabled algorithms for nucleotide sequence analysis to scale to unprecedented levels (e.g., Chikhi et al. 2024; Karasikov et al. 2025).

In addition to randomizing the input data, a hash function is often used to reduce its size; for example, a hash function such as MD5 could map a whole genome to a 64-bit integer. By reducing data size, hash functions can reduce the runtime and memory usage of downstream algorithms. By their nature, these hash functions will sometimes have collisions; that is, different input elements will map to the same hash value. In some situations, collisions may even be desired as a way to detect similar input elements.

Despite the wide use of hash functions in nucleotide sequence analysis, there is no comprehensive review of the types of hash functions used and their various applications. There has been some tangential coverage in other surveys, such as reviews on data structures for *k*-mer sets (Marchet et al. 2020; Chikhi et al. 2021) and reviews on sketching techniques in bioinformatics (Marçais et al. 2019b; Rowe 2019; Zheng et al. 2023). There are also books (Knuth 1968; Leskovec et al. 2020) and surveys (Chi and Zhu 2017; Jafari et al. 2021; Wu et al. 2022) covering hash functions in a broader context, but they do not review the specific peculiarities and applications in nucleotide sequence analysis.

As hash functions and hash-based algorithms have been at the core of progress in genomics, it is crucial to establish a unified framework that categorizes all these methods, highlights their properties, and identifies commonalities and differences. We provide a comprehensive overview of the hash functions applied and designed for nucleotide sequence analysis, filling a gap in the current literature. We note our focus is on the hash functions themselves rather than on an exhaustive list of their applications, though we do provide illustrative examples of applications throughout. We also note that though there is a close relationship between hash functions and sketches/embeddings, this survey focuses on hash functions. In particular, we take the perspective that for a hash function, there is no notion of distance between outputs; the outputs are treated as atomic values and the only meaningful comparison that can be made between them is to either check if they are equal or if one is less than the other.

## Overview

In this section, we will formally introduce hash functions and give a high-level overview of the types of hash functions that will be covered in the rest of the paper.

The section titled “ASCII-to-integer encoding” covers a basic but often poorly-described step of most hash functions. A DNA sequence is at first usually stored in the ASCII format (the same one used for text files), using the DNA characters {A, C, G, T}. Although convenient for human interpretation, it is unnecessarily wasteful to represent each character with a whole byte. Before applying more sophisticated hash functions, it is therefore common to encode the sequence compactly using two bits per nucleotide. The section will cover the various ways that this has been done in practice.

A hash function *h* is a function that maps an element from a universe *U* to an integer in [*B*] called a bucket (the notation [*B*] means the integers between 0 and *B* − 1). For example, *U* might be the set of all *k*-mers and *B* might be 2^32^. A hash function is *collision-free* if no two elements map to the same value; that is, the fact that *h*(*x*_1_) = *h*(*x*_2_) always implies that *x*_1_ = *x*_2_. A collision-free hash function is *practically invertible* if there exists an efficient algorithm that takes a bucket *b* and either finds the unique *x* such that *h*(*x*) = *b* or reports that no such *x* exists. Note that if there is a way to enumerate the elements of *U*, then a brute-force algorithm can find an inverse by just checking every element of the universe; hence, the key to being practically invertible is to be able to do it efficiently. Table 1 further summarizes the notation and definitions we will be using in this paper.

**Table 1.** Notations used in the paper

Notation	Meaning	Example
Σ	the DNA alphabet: {A, C, G, T}	
*S*	a sequence using Σ	*S* = AGTTC
*m*	length of *S*	*m* = 5 for *S* = AGTTC
*k*-mer	a sequence of length *k*	ACG (for *k* = 3)
*n*	number of *k*-mers in *S*: *m* − *k* + 1	*n* = 3 for *S* = AGTTC
*S*[*i*]	the *i*th character of *S*	*S*[1] = A for *S* = AGTTC
*s*_*i*_	the *k*-mer starting at the *i*th position of *S*	*s*_2_ = GTT for *S* = AGTTC
⊕	the “exclusive or” (XOR) operation	110 ⊕ 100 = 010
≫, ≪	shift right and shift left operations	100101 ≫ 2 = 001001
and	bitwise “and” operation	110 and 100 = 100
rol^*d*^(*x*)	*d* cyclic left rotations of *x*	rol^2^(100100) = 010010
*u* · *v*	dot product of *u* and *v*	(1, 0, 1) · (1, 1, 0) = 1 + 0 + 0 = 1
*J*( · , · )	the *Jaccard* similarity: $\frac{\left|X\cap Y\right|}{\left|X\cup Y\right|}$	*X* = {*a*, *b*, *c*}, *Y* = {*c*, *d*}
		$J(X,Y)=\frac{\left|\{c\}\right|}{\left|\{a,b,c,d\}\right|}=\frac{1}{4}$
[*n*]	the integers between 0 and *n* − 1	
*h* : *U* → [*B*]	a hash function from a universe *U* to the integers [*B*]	$h(x)=(2x+1)\mathrm{mod}2^{4}$
*domain* of *h*	the set *U*	all 64-bit integers
*codomain* of *h*	the set [*B*]	all 4-bit integers
*image* of *h*	{*h*(*x*)|*x* ∈ *U*}	all odd 4-bit integers
π : *U* → [|*U*|]	a permutation: a bijective hash function	π(x)=x⊕M, for 0 ≤ *M* < |*U*|, where |*U*| is a power of 2
{*h*_*θ*_|*θ* ∈ Θ}	a family of hash functions with seeds chosen from Θ	
sim( · , · )	a similarity measure associated with a metric space	*s*(*X*, *Y*) = *J*(*X*, *Y*)
*spaced word*	a subsequence extracted using a mask pattern	for mask 10011 and *S* = AGTTC, the extracted spaced word is ATC

Because a hash function follows the mathematical definition of a function, it is, strictly speaking, deterministic. In other words, for a given input, the output remains the same every time the function is run. In order to add randomness, we need to consider families of hash functions instead. If *H* is a family of hash functions, then by choosing *h* ∈ *H* uniformly at random, the user injects randomness into the hash process. For example, we might informally speak of a hash function that assigns an element to a bucket uniformly at random by flipping a *B*-sided coin for each element. But, formally, we would define a family of hash functions *H*_ideal_ which contains *B*^|*U*|^ deterministic hash functions. Each hash function corresponds to a particular instantiation of the |*U*| coin flips. We would then pick a hash function uniformly at random from *H*_ideal_. Although it is common to think of the hash function itself being random, the formal definition using families is needed for more nuanced understanding.

Hash families are often parameterized by a *seed*. For *H*_ideal_, the seed is the instantiation of the |*U*| coin flips. For another example, let h(x)=x⊕M be a simple hash function that XORs (see Table 1 for the definition of XOR) the input integer with another integer *M*, where 0≤M<|U| and for simplicity we assume that |*U*| is a power of 2. *M* is said to be a seed of this hash function, and we might even make it explicit by writing *h*_*M*_(*x*). More generally, a seeded hash family is defined as {*h*_θ_|*θ* ∈ Θ}, where Θ is the set of possible seed values (e.g., [|U|]) and *θ* is a particular seed (e.g., *M*). The user can then choose a hash function from this family uniformly at random by picking a uniformly random value for the seed.

**Figure 1.**
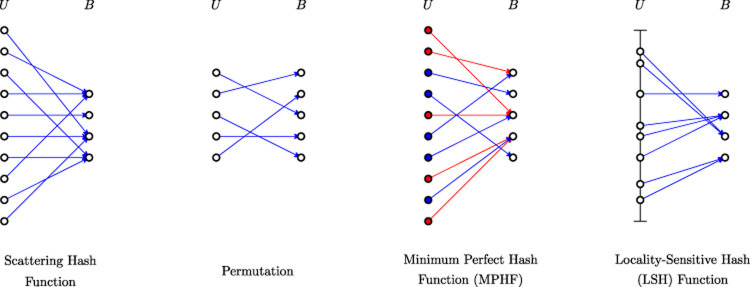
Illustration of the four categories of hash functions discussed in this survey. For scattering hash functions, there are many collisions in *B*. For permutations, there are no collisions. For the minimum perfect hash function (MPHF), blue dots represent the subset of the universe provided in advance, with red dots representing the rest. The blue edges should not have any collisions, but the behavior of the red edges is undefined. For the locality-sensitive hash function (LSH) category, the vertical line segment represents the metric space on which the universe elements lie, and elements with a small distance are more likely to have the same hash value. For this category, collisions are desired for elements that are nearby in *U*.

We classify the hash functions in this survey into four categories (see Fig. 1 for an illustration), which form the basis for the structure of this survey. The first category is what we refer to as *scattering hash functions*, following their original usage in computer science as a way to scatter elements into a hash table (Knuth 1968). In this situation, the universe *U* is huge; for example, for all possible 60-mers, the size of the universe *U* is 4^60^. The number of buckets *B* is typically much smaller, for example, 2^32^. As a result, scattering hash functions have hash collisions. Because collisions typically have adverse downstream effects, good scattering functions minimize the chance of collisions as much as possible.

The second category of hash functions is permutations. A hash function is a *permutation* when it is bijective; that is, it is collision-free and |*U*| = *B*. Unlike a scattering hash family, a permutation is not intended to be a way to place elements of *U* into a small number of buckets. Instead, the permutation serves to create a randomized total order on the elements of *U*. Permutations may be practically invertible, but not necessarily.

The third category is what we call *minimum perfect hash functions* (MPHFs). Scattering hash functions and permutations are generally chosen independently of the data set to which they will be applied. They might be chosen just based on the universe size and the desired number of buckets, but without any assumptions on which elements of the universe will end up being hashed. An MPHF, on the other hand, is constructed based on knowing the subset of the universe which will have a hash value assigned. This knowledge allows a hash function to be collision-free on the subset, though at the expense of needing more space to store the hash function.

The fourth category of hash functions is locality-sensitive hash (LSH) functions. Unlike the previous categories, these hash functions are designed to exploit collisions, rather than avoid them. Specifically, they assume that there is a reasonable notion of distance between elements in the universe, for example, the Hamming distance between two sequences. They then hash elements from the universe such that two elements with a small distance are more likely to collide than elements with a large distance. Unlike the other categories, LSH functions not only detect equal elements but detect similar elements.

## ASCII-to-integer encoding

For our purposes, an *encoding* is an invertible function from an ASCII DNA sequence *S* of length *m* to a bitvector of length 2*m*, or, equivalently, to an integer in [4^*m*^]. In this section, we do not consider non-DNA characters such as “N.” The natural approach for encodings is to assign a 2-bit code to each nucleotide. Given any 2-bit encoding of the nucleotides, the 2-bit encoding of a DNA sequence *S* is then obtained by concatenating the 2*m* bits that represent its constituent characters.

There are 4! = 24 options for a 2-bit encoding of nucleotides. A common one assigns A↦00, C↦01, G↦10, and T↦11. For example, the encoding of ACGG would be 00011010. This encoding has the sometimes desirable property that the lexicographical order of the ASCII sequence corresponds to the lexicographic order of the encoded bitvector. For example, the encoding of CCGT (i.e., 01011011) is larger, both as a string and an integer, than the encoding of ACGG, agreeing with the fact that CCGT is lexicographically larger than ACGG. This encoding also possesses a desirable property for implementation: given a character *c* ∈ Σ encoded in ASCII, the simple expression (3and((c≫2)⊕(c≫1))) provides its 2-bit encoding described above. This can be used to compute the encoding of a longer string using fast arithmetic operations, avoiding slow branching instructions. Furthermore, the expression works also for the four characters in lower case, due to the design of the ASCII code.

Another mapping swaps the encoding of *G* with that of *T* (i.e., A↦00, C↦01, G↦11, and T↦10). It can be computed with an even simpler expression (3and(c≫1)); it can also be used to save a bit for the canonical encoding (see below). Note that both aforementioned expressions to convert from an ASCII character to a 2-bit encoding work for the RNA alphabet {A, C, G, U} as well.

Other nucleotide encoding schemes are also used, depending on the applications. A 3-bit encoding can accommodate additional letters in the alphabet, such as N for the ambiguous nucleotides. A 1-bit encoding, for example, mapping purines (A and G) to 0 and pyrimidines (C and T) to 1, not only reduces the memory footprint but can also simplify biological analysis when the actual nucleotides are insignificant. *One-hot* encoding is another encoding scheme, popular in machine learning. It treats the alphabet Σ as categories and maps each character of a sequence *S* to a binary (column) vector of size |Σ|. For example, $A\mapsto 1000^{T}$, $C\mapsto 0100^{T}$, $G\mapsto 0010^{T}$, and $T\mapsto 0001^{T}$. The sequence *S* is therefore encoded as a |Σ| × *m* binary matrix. Because one-hot encoding is widely used in the field of machine learning to represent categorical input, applying it to DNA sequences facilitates the direct utilization of existing models for bioinformatics tasks.

Owing to the double-stranded nature of DNA, it is often desirable to identify a DNA sequence with its reverse complement. That is, if *S* and *S*′ are reverse complements, we would like their encodings to be the same. Encodings with this property are said to be canonical. This is a slight abuse of notation, as a canonical encoding is not invertible, as required by our definition of encoding; however, in the context of a canonical encoding, we interpret invertible to mean that we can recover the pair {*S*, *S*′}. The simplest canonical encoding is to encode the lexicographically smaller sequence between *S* and *S*′. For example, to encode CCGT, we observe that CCGT is larger than ACGG, and then use a standard encoding for ACGG. The same idea carries over to any hashing method: we compute the hash values of *S* and *S*′, then choose the smaller one as the final result (e.g., as is done by Zentgraf and Rahmann 2020).

Applying these canonical encodings roughly halves the size of the codomain: for encoding sequences with an odd length *m*, only 2^2*m*−1^ out of 2^2*m*^ output values are possible—the other half of the image is wasted. A simple trick can save one output bit by reducing the size of the image to match the codomain. This idea was first described in published form by Martayan et al. (2024) but had been already used in practice prior to that. Consider the base 2-bit encoding with A↦00, C↦01, G↦11, and T↦10 and assume that the sequence length *m* is odd. The *parity* of a bitvector is whether the number of 1s is odd or even. Observe that for each pair of complementary nucleotides, the parities of the base encodings are opposite. Therefore, the parities of the base encoding of *S* and *S*′ are also opposites. Our canonical encoding of *S* is created by (1) computing the base encodings of *S* and *S*′, (2) choosing the unique one that has an odd parity, and (3) dropping its higher-order (i.e., leftmost) bit. For decoding, the higher-order bit can be restored by checking the parity — the bit is 1 if the parity is even and 0 otherwise. For example, the base encodings of ACG and CGT are 000111 and 011110, respectively. The canonical encoding of {ACG, CGT} is then the base encoding of ACG (as it has odd parity) without the leftmost bit, that is, 00111. To decode 00111, we observe that it has an odd parity, and so we add a leftmost 0 bit and recover 000111. This type of canonical encoding only works when *m* is odd; for the case of even *m*, Wittler (2023) gave a more involved encoding scheme which we do not cover here.

Suppose that instead of encoding arbitrary sequences, the user is sliding a window along a (long) sequence *G* (e.g., a genome) with length *m* > *k* and encoding the *k*-mer of every window. Computing the encoding for each *k*-mer will take Θ(*k*) time if done naively using the above approaches (though it can be sped-up using specialized low-level hardware instructions). An encoding is said to be *rolling* if it can be computed in constant time from the encoding of the preceding *k*-mer. With a rolling encoding, the run time to compute all the encodings from *G* is reduced from Θ(*mk*) to Θ(*m*). The above base encodings can all be done in a rolling way as follows. The first *k*-mer in *G* can be encoded as usual. The *k*-mer in the next window can be encoded by taking the encoding of the first *k*-mer, dropping the higher-order (i.e., leftmost) two bits, and adding two lower-order (i.e., rightmost) bits which correspond to the encoding of the new nucleotide. For example, consider again the 2-bit encoding A↦00, C↦01, G↦11, and T↦10. Let *k* = 3 and *G* = ACGT. Then, the encoding of the first *k*-mer (i.e., ACG) is 000111. To obtain the encoding of the second *k*-mer (i.e., CGT), we drop the leftmost two bits to get 0111, and add the 2-bit encoding of T (i.e., 10) to the right, obtaining 011110. The encoding of the reverse complement of the second *k*-mer can be obtained from the encoding of the reverse complement of the first *k*-mer in a symmetric manner.

## Scattering hash functions

In this section, we will first describe what are the desirable properties of a scattering hash function and how they are evaluated. We will then give two examples of general-use scattering functions that are popular in genomic sequence analysis. Finally, we will describe a scattering function designed specifically for the case of genomic sequences.

The ideal scattering hash family is the aforementioned *H*_ideal_, which flips a *B*-sided coin for each element. *H*_ideal_ is often informally referred to as the “truly random” or “completely random” hash function. Unfortunately, using *H*_ideal_ is impractical because, even though computing the bucket of a *k*-mer *x* is fast, the bucket needs to be stored so that future queries for *x* consistently return the same bucket. In other words, one needs to store which hash function was chosen from *H*_ideal_, of which there are *B*^|*U*|^ possibilities. A basic result from information theory states that at least log_2_ *X* bits are needed to store an element that is chosen uniformly at random from a universe of size *X* (Navarro 2016). Thus storing *H*_ideal_ requires $|U|\log_{2}B$ bits, which is prohibitive in most applications.

### Evaluation of scattering hash functions

Scattering hash functions are designed so that they share some important theoretical properties with *H*_ideal_, even if they cannot achieve all of them. As a rule of thumb, the more properties of *H*_ideal_ that a hash family mimics, the more space it takes to store and/or the longer it takes to compute. A lot of computer science research is thus focused on developing hash functions that achieve certain tradeoffs between computational resources and the closeness to *H*_ideal_. However, in hash functions developed for nucleotide sequence analysis, it is rare to see any formal proofs of the properties. Rather, it is often experimental evaluation of the desired properties that convinces practitioners that a hash function fits their needs. Moreover, many practical hash functions do not arise from the academic community and are not associated with publications; this has resulted in a lack of standard terminology and formal underpinnings for their empirical evaluation. In this section, we will cover some of the theoretical guarantees that have been used for nucleotide sequence analysis and connect them to how they have been empirically evaluated.

A key design element of scattering hash functions is speed. They are typically very fast, implemented using a handful of simple arithmetic operators such as XOR, addition, subtraction, and shifting. The focus of empirical speed evaluation is therefore on the absolute throughput (i.e., number of hashes computed per second) rather than on asymptotic speed analysis.

One of the more basic guarantees of *H*_ideal_ is that an element is equally likely to get assigned to any bucket. Formally, there are two ways to interpret this property, depending on whether we assume that the hash function is fixed and the randomness comes from the data, or vice versa. In the first interpretation, we assume a single, deterministic hash function. The property is then called *regularity*: for every bucket *b*, if we draw an element from the universe uniformly at random, then the probability of it hashing to *b* is 1/*B*. For example, a hash function that returns the encoding of a *k*-mer modulo *B* is regular when |*U*| is a multiple of *B*. When proving the regularity of a function is too challenging, the property can be tested empirically by looking at the distribution of bucket sizes after hashing a large number of uniformly chosen elements (Kazemi et al. 2022 has a good example in their Fig. 1B). The limitation of this approach is that it does not reflect the fact that in nucleotide sequence analysis, input data is not random and hence may elicit poor behavior even if the function is regular. In the above example, if in practice all the data is a multiple of *B*, then all the data will collide in a single bucket.

The second formal interpretation is that for every element *x* and every bucket *b*, if we draw a hash function uniformly at random from a hash family, then the probability that *x* hashes to *b* is 1/*B*. This property is called *uniformity*. Empirically, one can fix an element and look at the distribution of the bucket sizes after hashing it using a large number of hash functions from the family. However, as the number of elements in the universe is typically huge, it is impractical to do such a test for a nonminuscule fraction of elements.

Although uniformity is desirable, it is not alone a sufficient guarantee for most applications. For example, a hash family may be such that it always places AAAA and CCCC into identical buckets. If that bucket is chosen using a coin flip, then the hash family is uniform. However, it is not independent. Formally, given an element *x*, we can view *h*(*x*) as a random variable, where the randomness comes from choosing *h* uniformly at random from the hash family. Then, a hash family is said to be *independent* if for any set of elements *x*_1_, …, *x*_*t*_, the buckets *h*(*x*_1_), …, *h*(*x*_*t*_) are independent. Independence is a very strong property, rarely achieved even in theory. A weaker but more achievable property is *universality*, which states for all choices of distinct elements *x*_1_ and *x*_2_, the probability of a collision is at most 1/*B*. An independent family is universal but a universal family is not necessarily independent.

An empirical benchmark used for evaluating scattering hash functions is SMHasher (Appleby 2025b) with its various tests described in a GitHub repository (Urban 2025). Independence and universality are difficult to evaluate empirically because the universe is too large to do exhaustive experiments. The SMHasher benchmark instead is built on the knowledge of how practical hash functions are often constructed; that is, it uses prior knowledge on the modes by which the independence guarantee might be violated by the functions it tests. For example, one of the tests is for the avalanche property (Chi and Zhu 2017; Upadhyay et al. 2022). Informally, the *avalanche property* states that by changing one bit in the input element, the hashed value should flip approximately half the bits. When viewed through the lens of independence, this property is testing whether there is dependence between certain pairs of input elements; that is, knowing *h*(*x*_1_) should not aid in knowing anything about *h*(*x*_2_), where *x*_2_ is obtained by flipping a bit of *x*_1_. If the avalanche property does not hold, then we know that *h*(*x*_2_) has a Hamming distance to *h*(*x*_1_) that is sufficiently different from half the number of bits. This contradicts independence, because if *h*(*x*_1_) were independent of *h*(*x*_2_), then the expected Hamming distance between them would be half the bits. Similar flavors of tests are done to test dependencies within certain groups of elements that are predisposed to dependence (e.g., differential tests and keyset tests).

### MurmurHash

There are several general-purpose scattering functions which are used for nucleotide sequence analysis. For example, xxhash (Collet 2025) is used to bucket *k*-mers by Shibuya et al. (2022) and CityHash (Pike and Alakuijala 2025) is used for sketching by Groot Koerkamp and Pibiri (2024). In this section, we will highlight the most widely used function, MurmurHash (Appleby 2025a,b). The original version of MurmurHash was posted online in 2008 (Appleby 2025a), and the C++ source code of different versions is available on GitHub (Appleby 2025b). It can take input values of any size, and generate a hash value of either 32-bit, 64-bit, or 128-bit length. We are not aware of any theoretical results on the uniformity or independence of MurmurHash. Empirically, its regularity has been tested by Abarca (2025), and it has been thoroughly tested using the SMHasher benchmark (Appleby 2025b).

A common approach to designing general-purpose scattering functions is to partition the input into chunks and perform a series of bitwise XORs, rotations, additions, and multiplications in order to scramble the input value. To give a concrete example, we will describe the 32-bit-output and ℓ-byte-input version of MurmurHash3 (Appleby 2025c). For simplicity we will assume that ℓ is a multiple of four. MurmurHash3 relies on five hard-coded 32-bit integer constants which, for the purposes of clarity, we will abstract away as *c*_1_, …, *c*_5_. It first partitions the input *x* into 32-bit blocks. For each 32-bit block, let *x*^*i*^ denote the value of the *i*th (indexed from 1 to *n*) block and let *h*^*i*^ denote a hash computed from *h*^*i*−1^ and *x*^*i*^. A special 32-bit value *h*^0^ serves as the seed. The hash values of *h*^*i*^ are then calculated recursively using the formula:

$$h^{i}=5\times {\mathrm{rol}}^{13}(h^{i-1}⊕(c_{2}\times {\mathrm{rol}}^{15}(c_{1}\times x^{i})))+c_{3}.$$

We have used × to indicate multiplication of 32-bit integers and rol^*d*^(*y*) to indicate *d* cyclic left rotations of *y*. The value of *h*^*n*^ is then transformed as follows. First, $h^{n}\leftarrow h^{n}⊕\ell$, then $h^{n}\leftarrow (h^{n}⊕(h^{n}\gg 16))\times c_{4}$, then $h^{n}\leftarrow (h^{n}⊕(h^{n}\gg 13))\times c_{5}$, and finally, $h^{n}\leftarrow h^{n}⊕(h^{n}\gg 16)$. The final return value *h*(*x*) is this transformed *h*^*n*^. To the best of our understanding, the choice of constants and operations in this definition is the result of a studied intuition and extensive trial and error. We stress that the five constants *c*_1_, …, *c*_5_ are hard-coded and are not seeds; the seed is *h*^0^.

MurmurHash has applications across many areas of nucleotide sequence analysis, including sequence alignment (Zaharia et al. 2011) and *k*-mer bucketing (Pandey et al. 2017). For example, Edgar (2021) applied it as the score function of short *s*-mers in the construction of syncmers. In the minmer winnowing scheme of Kille et al. (2023), it is used as the hash function for *k*-mers in the MinHash framework. MurmurHash also often serves as the basic hash function in Bloom filters (Patgiri et al. 2023), a popular data structure for storing and querying the presence of *k*-mers (Marchet et al. 2020).

### Karp-Rabin

The downside of MurmurHash is that it is not a rolling hash function. Let *s*_*i*_ be the *k*-mer starting at position *i* of a sequence *S*. In a rolling scenario, we are sliding a window across *S* and would like to compute *h*(*s*_*i*+1_) given that we have already computed *h*(*s*_*i*_). A hash function like MurmurHash requires time to process the whole *k*-mer. Instead, a rolling hash function is one that allows to compute *h*(*s*_*i*+1_) from *h*(*s*_*i*_) in constant time.

The classical way to do this is with the Karp-Rabin hash family (Karp and Rabin 1987). Let *x* be a *k*-mer and let *σ* be the size of the underlying alphabet, for example, *σ* = 4 for DNA or RNA sequences. Let enc(⋅) be a function encoding each alphabet character to a unique integer between 0 and *σ* − 1, for example, enc(′′A′′)=0, enc(′′C′′)=1, enc(′′G′′)=2, and enc(′′T′′)=3. Given a seed *p* which is a prime integer, the Karp-Rabin hash function (sometimes referred to as a Karp-Rabin fingerprint) is defined as

$$h_{\;p}(x)\;:=\left(\sum_{\;j=1}^{k}\sigma^{k-j}\mathrm{enc}(x[j])\right)\mathrm{mod}p.$$

One can gain the intuition behind *h*_*p*_ if we ignore the effect of *p* and consider an alphabet of base-10 digits whose encoding are given by enc(′′0′′)=0, …, enc(′′9′′)=9. Then,

$$h_{p}(\prime \prime 394\prime \prime )=\sum_{\;j=1}^{3}\sigma^{3-j}\mathrm{enc}(x[j])=100\cdot \mathrm{enc}(\prime \prime 3\prime \prime )+10\cdot \mathrm{enc}(\prime \prime 9\prime \prime )+1\cdot \mathrm{enc}(\prime \prime 4\prime \prime )=300+90+4=394.$$

The Karp-Rabin hash family is defined with respect to a positive integer *P*. Note that *P* is not a seed but defines the hash family itself. The hash function *h*_*p*_ is chosen from this family by picking a prime number *p* uniformly at random in the range between 2 and *P*. We are not aware of any formal result about the independence properties of this hash family, for example, whether it is universal.

Given a sequence *S*, one can compute *h*_*p*_(*s*_*i*+1_) from *h*_*p*_(*s*_*i*_) using the following formula:

(1) $$h_{p}(s_{i+1})=(\sigma h_{p}(s_{i})-\sigma^{k}\mathrm{enc}(S[i])+\mathrm{enc}(S[i+k]))\mathrm{mod}p.$$

To see this intuitively, consider *S* = ″3942″ with *k* = 3, while ignoring the effect of *p*. The formula states that

$$h_{p}(s_{2})=10h_{p}(s_{1})-10^{3}\mathrm{enc}(S[1])+\mathrm{enc}(S[4])=10(394)-3000+2=942.$$

More generally, one can use elementary number theory to show that Equation 1 holds even when accounting for doing the modulo operation.

Computing *h*_*p*_(*s*_*i*+1_) from *h*_*p*_(*s*_*i*_) takes a constant number of operations, independent of *k*. Furthermore, when *σ* = 4, the operations can be efficiently implemented using fast shifts and additions.

The choice of *P* controls the chance of collision. Observe that when *p* ≥ 4^*k*^, then the Karp-Rabin fingerprint of a DNA *k*-mer is simply its 2-bit encoding, meaning that it is collision-free. For smaller values of *p*, the probability of collision increases as *p* decreases. Therefore, setting *P* larger decreases the collision probability, at the expense of requiring more space (i.e., $\log_{2}P$) to store the fingerprint.

The above technique computes hash values from *k*-mers generated from a sliding window along *S*. In some situations, the user needs to compute hash values for spaced words generated in a similar manner. A *spaced word* is defined by a binary pattern (called a mask) where “1” indicates positions to be extracted and “0” represents positions to be ignored (Califano and Rigoutsos 1993). For example, a pattern “110101” applied to *S* = ACGTCA generates the spaced word ACTA. A spaced word can be encoded using the same concatenation of 2-bit nucleotide encodings as with *k*-mers, ignoring the masked locations. (More advanced encoding that are faster to compute are described by Harris 2007.) In this scenario, we would like to generate the hash of the spaced word starting at location *i* + 1 given the value at location *i*. Girotto et al. (2018b) implemented a variant of Rabin-Karp hashing that works for spaced words, which was further sped up by Girotto et al. (2018a), Petrucci et al. (2020) and Mian et al. (2024); we omit the details here. Mian et al. (2024) showed how the fast hashing algorithm can be integrated into a metagenomic read classifier (Clark-S, described by Ounit and Lonardi 2016) to get substantial speedups.

### ntHash

ntHash (Mohamadi et al. 2016) is a rolling hash family designed specifically for DNA/RNA sequences and based on the idea of cyclic polynomial hash functions (Cohen 1997). The seed to ntHash is a lookup table *T* that assigns a 64-bit integer to each nucleotide. The hash value of a *k*-mer is the 64-bit integer defined as

$$h_{T}(x)\;:={\mathrm{rol}}^{k-1}(T[x[1]])⊕\cdots ⊕{\mathrm{rol}}^{0}(T[x[k]]).$$

**Figure 2.**
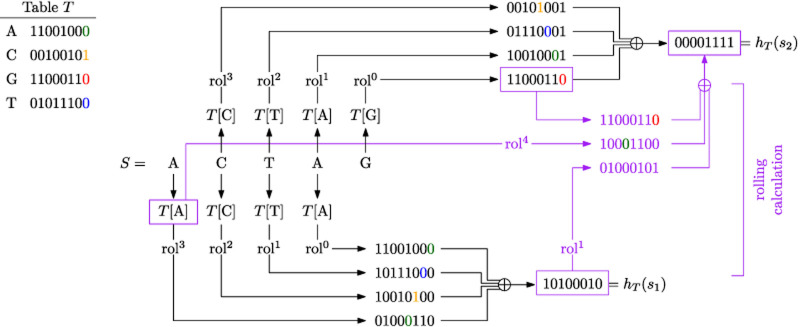
An illustration of ntHash with *k* = 4. The random table *T* maps each nucleotide to an 8-bit integer (for simplicity, we use 8 bits for the output, instead of 64 bits). A highlighted digit in a binary number indicates its trailing digit before applying any cyclic left rotation.

A hash function *h*_*T*_ from the ntHash hash family is selected by choosing *T* uniformly at random. For a sequence *S*, ntHash computes the hash value *h*_*T*_(*s*_*i*+1_) using *h*_*T*_(*s*_*i*_) using the formula (see Fig. 2 for an illustration)

$$h_{T}(s_{i+1})={\mathrm{rol}}^{1}(h_{T}(s_{i}))⊕{\mathrm{rol}}^{k}(T[S[i]])⊕T[S[i+k]].$$

This update operation takes constant time (i.e., independent of *k*) and can be implemented using fast XOR and shift instructions. A similar update formula allows one to simultaneously compute the hash of the reverse complement; this allows native support for canonical hash values by taking the minimum of the hashes of a *k*-mer and its reverse complement. When applied in a rolling setting, ntHash is substantially faster than MurmurHash while maintaining similar levels of empirical “randomness” to *H*_ideal_ (Mohamadi et al. 2016).

ntHash2 (Kazemi et al. 2022) builds upon ntHash in several ways. First, it changes the way that canonical hash values are computed by reporting the sum of the noncanonical values (modulo 2^64^), rather than the minimum. Second, it adds support for hashing of rolling spaced words. Third, it addresses some undesirable nonindependence properties of ntHash by replacing the rol operation with a “split rotation” operation instead.

ntHash and ntHash2 are applied widely in nucleotide sequence analysis, especially in rolling settings. In minimizer-based *k*-mer indexing, they generate canonical hash values for adjacent *k*-mers, with the smallest value selected as the window minimizer (Coombe et al. 2021; Rautiainen and Marschall 2021; Cracco and Tomescu 2023; Groot Koerkamp and Martayan 2025). They also support simultaneously computing multiple hash values for the same *k*-mer, without repeating entire calculations. This makes them widely adopted when Bloom filters are used; for example, they are used for de Bruijn graph construction in genome assembly (Jackman et al. 2017; Nip et al. 2020; Wong et al. 2023a).

## Permutations

In this section, we will describe the second category of hash functions – permutations. Permutations are often denoted using *π* instead of *h*, where *π*(*x*) returns the rank of *x* in the ordering specified by *π*. We assume that the input nucleotide sequence has already been encoded into a *w*-bit integer, so that the output is also a *w*-bit integer, that is, *U* = [2^*w*^] and *B* = 2^*w*^. The ideal permutation is one that is chosen uniformly at random from the family of all permutations. As there are |*U*|! possible permutations, storing a uniformly chosen one requires at least $\log_{2}(2^{w}!)=\Theta (w2^{w})$ bits. As with *H*_ideal_, this is generally prohibitive. Instead, practical permutations try to mimic the behavior of the ideal permutation family, while limiting space usage.

The evaluation of permutations is in many ways similar to that of scattering hash functions. One difference is that the ideal permutation family is not universal, because the joint probability that two distinct elements hash to the same value is 0 rather than 1/*B*. However, this kind of dependence has limited practical impact when *B* is large. As with scattering hash functions, the regularity, uniformity, and universality of permutations can be tested empirically using the SMHasher suite.

A property particular to permutations is min-wise independence. A permutation family is *min-wise independent* if for any subset *X*⊆ *U*, each element of *X* has an equal probability of having the smallest rank among all elements in *X*. This property is not tested by SMHasher and we have not come across instances of this property being tested empirically in the nucleotide sequence analysis literature. As we will see in later sections, this property is needed for some applications.

We note that uniformity does not imply min-wise independence. For example, there may exist two elements *x*_1_ and *x*_2_ such that the value of *π*(*x*_1_) is chosen uniformly at random but then the value of *π*(*x*_2_) is always set to $\left((\pi (x_{1})+1)\mathrm{mod}B\right)$. For any hash value *b*, both elements have an equal chance 1/*B* of hashing to *b* when considered independently, but, in *B* − 1 cases, the hash of *x*_2_ will be larger than that of *x*_1_. Thus, for the subset *X* = {*x*_1_, *x*_2_}, the probability that *x*_2_ has the smallest rank among the elements in *X* is 1/*B*, whereas the same probability for *x*_1_ is (*B* − 1)/*B*.

A simple, widely-used permutation family works by choosing, uniformly at random, a *w*-bit integer *M* called a mask (Wood and Salzberg 2014; Quedenfeld and Rahmann 2017; Greenberg et al. 2023). The permutation is then defined as $\pi_{M}(x)=x⊕M$. Observe that this is indeed a permutation because the XOR function is invertible, that is, (x⊕M)⊕M=x. It is also uniform, as for every *x* and every *b*, there is exactly one mask M=x⊕b that satisfies x⊕M=b. Hence, $\underset{M}{\mathrm{Pr}}[\pi_{M}(x)=b]=1/B$. However, *π*_*M*_ is very bad when it comes to independence. In fact, once one knows the hash value for a single element *x*, one can infer *M* and thereby know the hash values of every other element. Moreover, the hash values are correlated with input elements in potentially unacceptable ways. For example, consider the encoding A↦00, C↦01, G↦10, and T↦11. Let *x*_1_ and *x*_2_ be two *k*-mers whose only difference is that *x*_1_ ends in an A and *x*_2_ ends in a C. Regardless of the mask *M*, $x_{1}⊕M$ and $x_{2}⊕M$ will remain identical except the last order bit. If the goal of the permutation is to scatter elements so that similar *k*-mers are in different places, then *π*_*M*_ may be unacceptable. Furthermore, *π*_*M*_ is not min-wise independent; if *X* contains exactly one element *x* with the highest-order (i.e., leftmost) bit set to 0, then the probability that $\pi_{M}(x)=\min\{\pi_{M}(x^{\prime })\;|\;x^{\prime }\in X\}$ is 50%, even if |*X*| > 2.

There are several other permutation families that have been used in nucleotide sequence analysis. One of the earliest uses of permutations was by the Jellyfish *k*-mer counter (Marçais and Kingsford 2011). MurmurHash3 is collision-free when both the input and output are 32 bits, making it a permutation (Appleby 2025b). A slight modification of ntHash is also collision-free in such a scenario (Groot Koerkamp 2025a). The Ranhash hash function from Press et al. (2007) was used in KmerGenie (Chikhi and Medvedev 2013). Zentgraf and Rahmann (2020, 2022) use a permutation family $\pi_{a,b}(x)=a({\mathrm{rol}}^{w/2}(x)⊕b)\mathrm{mod}2^{w}$, where the family is defined over uniform choices of 0 ≤ *b* < 2^*w*^ and odd *a* in the range 0 ≤ *a* < 2^*w*^. Another permutation function is sometimes referred to as “Thomas Wang’s function” due to its author (Wang 2025) and is available as a code snippet in Li (2025). It is used in exact *k*-mer counting (Pandey et al. 2017) and mapping (Li 2018), among other places. Note that this is not a family but a single deterministic function. Another permutation family is fxhash (Breeden 2025), used by Groot Koerkamp (2025b), which simply multiplies the 64-bit input by a uniformly random 64-bit odd integer and takes the remainder. The fact that 2^64^ must be co-prime with any odd number guarantees that the function is invertible.

## Minimum perfect hash functions

The third category of hash function are minimum perfect hash functions (MPHFs). In some scenarios, there is a subset *U*′ of the universe that is known in advance and for which collisions are undesired. For example, *U* could be the set of all *k*-mers and *U*′ could be the set of *k*-mers in the human reference genome. A typical situation is storing some associated information with each *k*-mer in *U*′, for example, its count in a sequencing experiment. A scattering hash function can be used to construct a hash table to achieve this; however, in order to resolve collisions, one would need to store the *k*-mer sequences themselves in the hash table. This adds a large space overhead. However, it is possible to design a hash function *h* that uses the fact that *U*′ is known in advance to assign an integer in [|*U*′|] to each element, without collisions. Such a function is called a minimum perfect hash function. It is then possible to replace a hash table with a simple array (i.e., direct access table) and avoid storing the *k*-mer sequence for each element. Note that when *h* is applied to an element not in *U*′, the return value can be any integer in [|*U*′|]. Therefore, an MPHF cannot be used to determine whether an input element belongs to *U*′ or not.

In contrast to the other categories of hash functions, MPHFs are not typically evaluated for their “randomness” properties. Instead, they are evaluated for the space needed to store and the time needed to evaluate the function itself (which, unlike for scattering hash functions and permutations, are no longer trivial).

We are aware of at least four MPHFs used in nucleotide sequence analysis: CHM (Czech et al. 1992), BBHash (Limasset et al. 2017), PTHash (Pibiri and Trani 2021), and LPhash (Pibiri et al. 2023). All except LPhash are generic implementations, not specific to nucleotide sequences. LPhash on the other hand is designed specifically for *k*-mer sets and attempts to assign consecutive hash values to pairs of *k*-mers that overlap by *k* − 1 nucleotides. The techniques used to construct MPHFs rely on concepts from compact data structures (Navarro 2016) and other advanced *k*-mer techniques (Chikhi et al. 2021). We therefore do not go into detail of how they are designed and instead refer the reader to Lehmann et al. (2025) for a recent survey on MPHFs. In terms of applications, MPHFs form the core of broadly used *k*-mer dictionaries such as Pufferfish (Almodaresi et al. 2018) and SSHash (Pibiri 2022).

## Similarity estimators

The hash functions considered so far do not consider distances between elements in the universe. However, many critical applications in sequence analysis do need to deal with “similarity” or “distance” between sequences. For example, large-scale genomic/metagenomic analysis requires estimating the similarity of two genomes (Ondov et al. 2016). Another example is the construction of the overlap graph during genome assembly, where a critical step is identifying overlaps between long error-prone reads (Myers 2005). The overlaps between long reads are of high similarity, as they originate from the same region of the genome, but are not identical due to sequencing errors. A third example is analyzing sequencing reads that are tagged with short sequences called Unique Molecular Identifiers (UMIs). Reads that have UMIs that are within a small edit distance of one another and map to the same gene and cell are likely to originate from the same molecule (Bose et al. 2015; Smith et al. 2017). Therefore, reads with similar UMIs can be clustered together so as to enable the correction of their sequencing errors.

Such applications involve similarity or distance measures, which necessitate that the universe *U* be a metric space. A metric space is a set of elements equipped with a distance function, or equivalently in most cases, a similarity measure sim(· , ·). For example, points in two or three dimensions naturally use the Euclidean distance. Hamming distance is a common distance measures for sequences of equal length. For biological sequences, the unit-cost edit distance (also called the Levenshtein distance) is fundamental as substitutions and indels are basic evolutionary events.

We will see in the next section how such distance metrics give rise to a broad class of functions called locality-sensitive hash functions. In this section, however, we will focus on a special subclass that is of its own interest. Consider as above a universe *U* equipped with a similarity function sim(· , ·). A hash family *H* = {*h*_*θ*_|*θ* ∈ Θ} is a *similarity estimator* if for any pair *x*, *y* ∈ *U*,

$$\underset{h_{\theta }\in H}{\mathrm{Pr}}[h_{\theta }(x)=h_{\theta }(y)]=\mathrm{sim}(x,y).$$

Here, the notation $\underset{h_{\theta }\in H}{\mathrm{Pr}}$ is used to mean that the probability space is defined by choosing *h*_θ_ uniformly at random from *H*. In statistical terminology, these type of functions are sometimes referred to as unbiased estimators (Wasserman 2004). In this section, we describe several similarity estimators that exist for similarity measures used in nucleotide sequence analysis.

### Hamming similarity

Let *U* be the set of sequences of length *m* and let *x*, *y* ∈ *U*. The *Hamming similarity* is defined as the fraction of positions where *x* and *y* agree or, formally,

$$\mathrm{sim}(x,y)\;:=\frac{\left|\{1\leq i\leq m|x[i]=y[i]\}\right|}{m}.$$

Let *h*_*i*_(*x*) be the hash function that simply returns the *i*th letter of *x*, and let *H* = {*h*_*i*_| 1 ≤ *i* ≤ *m*} be the family with *m* such hash functions. Assuming that a hash function is drawn uniformly at random from *H*, it follows immediately that *H* is a similarity estimator for Hamming similarity:

$$\underset{h_{i}\in H}{\mathrm{Pr}}[h_{i}(x)=h_{i}(y)]=\sum_{i=1}^{m}\frac{1\{x[i]=y[i]\}}{m}=\mathrm{sim}(x,y).$$

Here, **1**{ · } is the indicator function, returning 1 if the condition inside is true and 0 otherwise.

### Angular similarity

Let *U* be the universe of real-valued *n*-dimensional unit vectors; formally, $U=\{u\in R^{n}|\|u{\|}_{2}=1\}$. Let ang(*x*, *y*) denote the angle between *x*, *y* ∈ *U*. The *angular similarity* between *x*, *y* ∈ *U* is defined as

$$\mathrm{sim}(x,y)\;:=1-\frac{\mathrm{ang}(x,y)}{\pi }.$$

The idea behind an estimator for angular similarity is *random projection*. Let $r\in R^{n}$ be real-valued *n*-dimensional vector. Define the hash function *h*_*r*_(*x*) as being 1 if the dot product between *x* and *r* is non-negative and 0 otherwise. We will consider the hash family *H* = {*h*_*r*_|*r*}, with the coordinates of *r* drawn independently at random from the standard Gaussian distribution. We now argue that *H* is a similarity estimator for the angular similarity. It is a classic result (Muller 1959) that $r/\|r{\|}_{2}$ is uniformly distributed on the unit *n*-sphere centered at the origin. Therefore, the event that *h*_*r*_(*x*) ≠ *h*_*r*_(*y*) is equivalent to the event that the hyperplane defined by *r* separates *x* and *y*. The latter happens with a probability of ang(*x*, *y*)/π, hence

$$\underset{h_{r}\in H}{\mathrm{Pr}}[h_{r}(x)=h_{r}(y)]=1-\frac{\mathrm{ang}(x,y)}{\pi }=\mathrm{sim}(x,y).$$

### Jaccard similarity

Let Ω be a set and let *X* and *Y* be two subsets of Ω. In our setting, Ω is usually a set of *k*-mers. The Jaccard similarity between *X* and *Y* is the fraction of their *k*-mers that are shared and is formally defined as

$$\mathrm{sim}(X,Y)\;:=\frac{\left|X\cap Y\right|}{\left|X\cup Y\right|}.$$

Let *π* be chosen uniformly at random from a family of min-wise independent permutations of Ω. Recall that π defines an order for all elements in Ω and π(*a*) denotes the rank of *a* in this order. The *set-min hash function*,^5^ introduced by Broder (1997), is a function *h*_π_(*X*) mapping *X* to the element in *X* with the smallest rank according to *π*. Formally, for a subset *X* ⊆ Ω,

$$h_{\pi }(X)\;:=\underset{x\in X}{\mathrm{argmin}}\pi (x).$$

Note that in our setting, the domain of the set-min hash function is the power set of Ω and not Ω itself. Let $H=\{h_{\pi }|\pi \;\mathrm{is}\;a\;\min-\mathrm{wise}\;\mathrm{independent}\;\mathrm{permutation}\;\mathrm{over}\;\Omega \}$ be the family of hash functions which chooses *π* uniformly at random. We now show that *H* is an estimator for Jaccard similarity. By definition of min-wise permutation, any element in X∪Y will have the equal probability of being the smallest element under *π*. Thus the event that *h*_π_(*X*) = *h*_π_(*Y*) happens if and only if the smallest element also falls in X∩Y. Hence, min-wise independence implies that

$$\underset{h_{\pi }\in H}{\mathrm{Pr}}[h_{\pi }(X)=h_{\pi }(Y)]=\frac{\left|X\cap Y\right|}{\left|X\cup Y\right|}=\mathrm{sim}(X,Y).$$

Min-wise independent permutation families are of theoretical interest (Broder et al. 1998), but in practice, *π* is often chosen using one of the options described in the “Permutations” section. In some instances, a scattering hash function is used and collisions are resolved using a tie-breaker.

### Sampling to reduce variance

The evaluation of the above similarity estimators focuses on their variance. Consider, for example, the set-min hash function applied to two sets with sim(*X*, *Y*) = 0.8. In practice, we pick *π* uniformly at random and check if *h*_π_(*X*) = *h*_π_(*Y*). For that specific *π*, the probability of a collision is some specific value, for example, 0.7. If we repeat this again for a different π, we would get a different collision probability, for example, 0.9. The theory says that on average, we will get probabilities of 0.8. However, in practice there is a big difference between seeing 0.7 and 0.9 versus seeing 0.79 and 0.81. The variance of an estimator captures this difference, that is, the extent to which the collision probabilities are concentrated around their mean values. A formal definition of variance is not needed here but the interested reader can check Wasserman (2004).

In order to improve variance, the above similarity estimators are often combined with sampling. For example, for the Jaccard similarity, one can select not just one π but many π, uniformly at random, and count the fraction of times that *h*_π_(*X*) and *h*_π_(*Y*) collide. As with the probability of a collision for a single π, this frequency of collisions is a similarity estimator of the Jaccard similarity of *X* and *Y*. However, the variance of the estimation decreases with more samples, at the expense of higher computational cost. This is also an example of how a hash function (e.g., set-min) can be sampled multiple times to form a sketch; that is, the set of computed *h*_π_(*X*) values is referred to as a MinHash sketch of *X* (Broder 1997). This idea has been extensively explored, resulting in new theories and practical tools for estimating similarity of large-scale sequencing data sets (Ondov et al. 2016; Pierce et al. 2019; Irber et al. 2022).

### Applications

In the next section, we will describe several methods for genomics applications that are built on top of these similarity estimators. Here, we will focus on the application of set-min. It has been widely applied in genomics over the past two decades, mostly as a basis for computing two types of sketches. We have already seen the first type of application, which is as a basis for the MinHash sketch, leading to a low-variance estimator for the Jaccard similarity between *k*-mer sets. MinHash sketches are used to estimate average nucleotide identity (Ondov et al. 2016), cluster metagenomes (Yang et al. 2011; Rasheed and Rangwala 2013), and classify sequences (Drew and Hahsler 2014). One can also define MinHash by using set-min with an underlying scattering function (i.e., with collisions), rather than a permutation; in such cases, there is a tradeoff between the number of bits used to store each hash value and the variance of the Jaccard estimator (Zhao 2019; Ertl 2021; Agret et al. 2022; Yu and Weber 2022; Baker and Langmead 2023; Xu et al. 2024). See Groot Koerkamp (2024) for a survey of this direction. Another extension of MinHash is to multi-sets of *k*-mers, called weighted MinHash (Wu et al. 2022); it has been applied to phylogenetic profiling (Moi et al. 2020), genome search (Zhao et al. 2024), and long read classification (Das and Schatz 2022).

The second type of application is the winnowed minimizer sketch (Schleimer et al. 2003; Roberts et al. 2004). Whereas in MinHash, many hash functions are drawn and applied to a single long string, in the winnowed minimizer sketch, only one hash function is drawn but it is computed for many short windows. In particular, set-min is applied to each sliding window of a long sequence, with each minimum *k*-mer referred to as a minimizer and the joint set of minimizers referred to as the winnowed minimizer sketch. This sketch is usually used as a seeding method for seed-and-extend sequence comparison such as genome assembly (Ekim et al. 2023), read mapping (Kille et al. 2023), read overlap detection (Li 2016), and sequence alignment (Li 2018). They are also utilized in de Bruijn graph indexing and querying (Holley and Melsted 2020) and *k*-mer counting (Deorowicz et al. 2015; Li and Yan 2015). See Ndiaye et al. (2024) for a more extensive survey of minimizer sketch applications.

## Locality-sensitive hash functions

Similarity estimators are a special case of a more broad category of hash functions. Let *U* be a metric space coupled with similarity measure sim( · , · ). A *locality-sensitive hashing (LSH) function* maps elements from *U* to buckets *B*, such that two distinct elements *x*, *y* ∈ *U* are more likely to collide (i.e., hash to the same bucket) if sim(*x*, *y*) is large, and less likely to collide if sim(*x*, *y*) is small. LSH functions formalize the idea of using the probability of collision to distinguish similar and dissimilar pairs of items.

Let *H* = {*h*_*θ*_|*θ* ∈ Θ} be a family of hash functions, where *h*_*θ*_ denotes a hash function *U* → [*B*]. Note that we can equivalently think of the hash function as mapping to a set of DNA strings of a fixed length of log_4_*B*. Given four parameters *s*_1_ ≥ *s*_2_ and *p*_1_ ≥ *p*_2_, we say that *H* is (*s*_1_, *s*_2_, *p*_1_, *p*_2_)-sensitive if for any pair of elements *x*, *y* ∈ *U*,

$$\begin{array}{rl} \mathrm{sim}(x,y) & \geq s_{1}\;\text{implies that}\;\underset{h_{\theta }\in H}{\mathrm{Pr}}[h_{\theta }(x)=h_{\theta }(y)]\geq p_{1},\;\mathrm{and}\\ \mathrm{sim}(x,y) & \leq s_{2}\;\text{implies that}\;\underset{h_{\theta }\in H}{\mathrm{Pr}}[h_{\theta }(x)=h_{\theta }(y)]\leq p_{2}. \end{array}$$

A similarity estimator, as defined in the previous section, is a special case of an LSH. Formally, a hash family *H* is a similarity estimator if and only if *H* is (*s*, *s*, *s*, *s*)-sensitive for every *s* ∈ (0, 1).

An important use of LSH functions is to improve scalability by organizing elements into “buckets” and thus avoiding exhaustive all-vs-all comparisons. For example, consider the problem of finding all pairs of sequences with edit similarity at least *s*_1_ in a given set of sequences. We can assign each sequence into a bucket using a (*s*_1_, *s*_2_, *p*_1_, *p*_2_)-sensitive LSH function for edit similarity. With high probability (i.e., *p*_1_), sequences with an edit similarity of at least *s*_1_ will be assigned to the same bucket. A second pass traverses individual buckets and computes the edit similarity for all pairs in each bucket to filter out false positive pairs (i.e., pairs with a similarity less than *s*_1_). Note that the all-vs-all computation is now limited to be within individual buckets rather than on the whole data set. The size of each bucket is expected to be small, as the probability that sequences with similarity less than *s*_2_ are assigned to the same bucket is low (i.e., *p*_2_). Other concrete uses of such bucketing strategies are in generating error-tolerant seeds for read mapping or detecting overlaps among large numbers of error-prone long reads.

In this section and the next, we will focus on LSH functions beyond similarity estimators. Some of these are not formally proven to satisfy the two properties above but are nevertheless designed to approximately exhibit the LSH behaviors; such *heuristic* LSHs use empirical evaluation rather than formal proofs. We will first present AND/OR amplification, a technique to boost the performance of LSH functions by increasing the separation between *p*_1_ and *p*_2_. We will then present an LSH for Hamming distance (spaced words) and a heuristic LSH for cosine similarity (SimHash).

### AND/OR amplification

LSH functions can be interpreted as mapping similar elements (i.e., similarity larger than *s*_1_) into the same buckets with (high) probability *p*_1_ *and* mapping dissimilar elements (i.e., similarity smaller than *s*_2_) into different buckets with (high) probability 1 − *p*_2_. We therefore can interpret *p*_1_ as sensitivity and *p*_2_ as false positive rate (1 − precision). This interpretation aligns perfectly with the need to tolerate errors in sequence analysis.

AND/OR amplification is a standard technique to improve the sensitivity (*p*_1_) and precision (1 − *p*_2_) of LSH functions, at the cost of speed. It has been adapted and applied in several places in nucleotide sequence analysis, some of which we describe in the following sections (i.e., MinHash, SimHash, and SubseqHash). As discussed above, for an (*s*_1_, *s*_2_, *p*_1_, *p*_2_)-sensitive family *H*, it is desirable to have large *p*_1_ and small *p*_2_. Amplification is a general approach that uses repeated sampling to improve these probabilities for any existing LSH family *H*.

For AND-amplification, consider sampling *r* hash functions *h*_1_, …, *h*_*r*_ from *H*, with replacement, and defining two elements to collide if and only if they have the same value under every *h*_1_, …, *h*_*r*_. Formally, we define a new hash function *h*′(*x*) := (*h*_1_(*x*), *h*_2_(*x*), …, *h*_*r*_(*x*)) and we consider *h*′(*x*) = *h*′(*y*) if and only if *h*_*i*_(*x*) = *h*_*i*_(*y*) for all 1 ≤ *i* ≤ *r*. The family *H*′ = {*h*′} is called an *r*-way *AND-construction*. If the probability of collision in *H* is *p*, then the probability of collision in *H*′ is *p*^*r*^. Therefore, *H*′ is $\left(s_{1},s_{2},p_{1}^{r},p_{2}^{r}\right)$-sensitive. AND-amplification improves precision because, because $p_{2}^{r}\leq p_{2}$, dissimilar elements are less likely to be hashed to the same value.

For OR-amplification, we can sample *b* functions from *H* and consider two elements to collide if and only if they collide on at least one of the *b* hash functions. The resulting family is called a *b*-way *OR-construction*. If the probability of collision for a single hash function is *p*, then the probability of collision in the OR-construction is 1 − (1 − *p*)^*b*^. Therefore, the OR-construction is (*s*_1_, *s*_2_, 1 − (1 − *p*_1_)^*b*^, 1 − (1 − *p*_2_)^*b*^)-sensitive. As 1 − (1 − *p*_1_)^*b*^ ≥ *p*_1_, similar elements are more likely to share a hash value, which improves sensitivity. In practice, the OR-construction is typically implemented by running LSH bucketing for *b* rounds, each using a newly sampled function from the original family *H*. The results of these rounds are then combined by taking their union to obtain all similar pairs.

OR-amplification can be generalized to define a collision between two elements if at least *b*′ collisions occur across the *b* sampled functions, where 1 ≤ *b*′ ≤ *b* is a predefined threshold. Although this mirrors the sampling technique used in similarity estimators to reduce variance, the objective here is different, that is, to tune the balance between sensitivity and precision without increasing the total number of sampled functions.

Perhaps surprisingly, the amplifications can be stacked to simultaneously increase both precision and recall. An *r*-way AND-construction followed by a *b*-way OR-construction produces an $\left(s_{1},s_{2},1-{\left(1-p_{1}^{r}\right)}^{b},1-{\left(1-p_{2}^{r}\right)}^{b}\right)$-sensitive family. One can show that as long as *p*_1_ is strictly greater than *p*_2_, there is always some combination of *r* and *b* that will result in the stacked construction both increasing *p*_1_ and decreasing *p*_2_. The improved accuracy, however, comes at the cost of efficiency, as one needs to evaluate each element using *r* · *b* hash values instead of one. For example, choosing *r* = 6 and *b* = 19, an (*s*_1_, *s*_2_, 0.7, 0.4)-sensitive family can be improved to an (*s*_1_, *s*_2_, 0.907, 0.076)-sensitive family at the cost of using 6 · 19 = 114 hash functions.

### Spaced words for hamming similarity

Recall that a spaced word is defined using a binary pattern called a mask. For example, if *b* = 110101 is the mask and *S* = ACGTCG, then the spaced word extracted from *S* using *b* is ACTG. The weight of a mask is the number of ones it contains (e.g., 4 in this example) and the length of a mask is the length of the pattern (e.g., 6 in this example). A spaced word can be viewed as a hash function *h*_*b*_, for example, *h*_*b*_(*S*) = ACTG. Similar to the winnowed minimizer sketch, it is applied to rolling windows along a long string; here, we will focus on the properties of applying it to a single window. In this subsection, we show that spaced words can be interpreted as locality-sensitive hash functions, despite not being commonly seen that way.

Let $H_{w}\;:=\{h_{b}|b\;\mathrm{is}\;a\;\mathrm{bitvector}\;\mathrm{with}\;\mathrm{length}\;m\;\mathrm{and}\;\mathrm{weight}\;w\}$^6^ be the set of hash functions induced by all spaced words of weight *w*, while keeping the length fixed. We now connect *H*_*w*_ to the LSH family *H* := {*h*_*i*_| 1 ≤ *i* ≤ *m*} for Hamming similarity that we described in the previous section. Clearly, picking a function *h*_*b*_ from *H*_*w*_ uniformly at random is equivalent to picking *w* indices *i*_1_, …, *i*_*w*_ and the corresponding hash functions, $h_{i_{1}},\ldots ,h_{i_{w}}$, without replacement at random from *H*. By the definition of *h*_*b*_, for any two length-*m* sequences *S*_1_ and *S*_2_, *h*_*b*_(*S*_1_) = *h*_*b*_(*S*_2_) if and only if *h*_*i*_(*S*_1_) = *h*_*i*_(*S*_2_) for every *i* ∈ {*i*_1_, …, *i*_*w*_}. Hence, *H*_*w*_ is similar to a *w*-way AND-amplification of the Hamming similarity estimator, with the only difference being that AND-amplification selects functions with, rather than without, replacement. With this caveat in mind, *H*_*w*_ can be approximately viewed as an (*s*, *s*, *s*^*w*^, *s*^*w*^)-sensitive LSH for all *s* ∈ (0, 1). In applications, an additional layer of OR-amplification is typically applied by using multiple masks to enhance the sensitivity of spaced words.

Spaced words were a major advancement in approximate sequence matching when they were popularized by PatternHunter (Ma et al. 2002) (the idea was first introduced much earlier by Califano and Rigoutsos 1993). PatternHunter achieves sequence comparisons twenty times faster than BLAST (Altschul et al. 1990, 1997) while using one-tenth the memory. Their applications then span multiple domains in bioinformatics. In homology search, spaced words have shown improvements in both speed and sensitivity (Brown et al. 2004; Hahn et al. 2016; Noé 2017). Sequence alignment tools have incorporated spaced words to improve candidate location identification. Modern aligners (David et al. 2011; Langmead and Salzberg 2012; Li 2018) use spaced words to identify potential matching regions with greater sensitivity than consecutive seed approaches. De novo assembly represents a novel application of spaced words. Assembly algorithms (Birol et al. 2015) use specially designed seed patterns with equal numbers of matching positions at the ends and contiguous gaps in the middle. This design reduces memory in de Bruijn graph construction while maintaining the ability to detect sequence overlaps. Metagenomic analysis has also benefited from spaced words. The approach enables efficient species identification in mixed samples through improved *k*-mer coverage estimation (Břinda et al. 2015).

### SimHash for cosine similarity

Angular similarity can be applied not just to real-valued vectors but also to sets. Consider two sets *S*_1_ and *S*_2_ with elements from the universe Ω. Let *u*(*S*) ∈ {0, 1}^|Ω|^ be the characteristic vector of *S*, with respect to a total order on Ω. That is, *u*(*S*) is a vector where each dimension corresponds to an element of the universe and has a binary value corresponding to the presence/absence of that element in *S*. One can define a similarity measure between two sets as the cosine of the angle between their characteristic vectors, that is,

$$\mathrm{sim}(S_{1},S_{2})\;:=\cos(\mathrm{ang}(u(S_{1}),u(S_{2}))).$$

This similarity measure has a nice interpretation in terms of the set intersection, by the following property:

$$\cos(\mathrm{ang}(u(S_{1}),u(S_{2})))=\frac{u(S_{1})\cdot u(S_{2})}{\left(\|u(S_{1}){\|}_{2}\cdot \|u(S_{2}){\|}_{2}\right)}=\frac{\left|S_{1}\cap S_{2}\right|}{\sqrt{\left|S_{1}|\cdot |S_{2}\right|}}.$$

This is similar to the Jaccard similarity but with a different denominator (i.e., the geometric mean of the set sizes).

We are not aware of any hash functions that are provably an LSH for the cosine similarity between sets. However, due to the close connection between cosine similarity and angular similarity, the similarity estimator for angular similarity (from the previous section) has been adopted to construct a heuristic LSH for cosine similarity over sets. Specifically, given a seed integral vector *w* ∈ {1, − 1}^|Ω|^, we define the hash function as:

$$h_{w}(S)\;:=\left\{ \begin{array}{cc} 1\; & \mathrm{if}\;u(S)\cdot w\geq 0\;\\ 0\; & \mathrm{otherwise}.\; \end{array}\right.$$

Intuitively, the above hash function conducts a majority vote among all elements in *S*: the hash value is 1 if more elements vote for 1 and the hash value is 0 if more elements vote for −1. The vector *w* determines which way each element votes.

SimHash is defined in Henzinger (2006) as the family of hash functions {*h*_*w*_} over all *w* ∈ {1, − 1}^|Ω|^. Note that because the direction of *w* is not uniformly distributed, as required by the angular similarity estimator, it is not obvious that SimHash is an LSH for the cosine similarity between sets. Nonetheless, SimHash has been widely used to compare web pages, texts, and recently biological sequences. In nucleotide sequence analysis, the set *S* is often the collection of all *k*-mers of an input sequence, in which case the universe Ω consists of all *k*-mers.

Owing to time and space considerations, SimHash is often used by first applying an internal hash function that maps each element of Ω to a *b*-bit integer, which corresponds to sampling *b* hash functions from the family {*h*_*w*_}. (The underlying hash function should ideally be the Cartesian product of *b* copies of *H*_ideal_ with *B* = 2 so that the sampling is uniform and independent. However, it is often approximated by general-purpose hash functions such as MurmurHash.) This transforms (the set *S* of) an input sequence into a set of *b*-bit integers, for which *b* majority votes are conducted among bits at the same position, thus obtaining a length-*b* binary vector as the hash value. Two sequences are considered similar if the Hamming distance between their *b*-bit SimHash values is at most *b*′, where 0 ≤ *b*′ < *b* is a predefined threshold. This corresponds to generalized OR-amplification, as discussed previously. A recent work named BLEND (Firtina et al. 2023b) extends this idea to identify similar, rather than just exactly matching, seeds among genomic sequences.

## LSH functions for the edit distance

Nucleotide sequences naturally undergo mutations over time. These mutations can be quantitatively estimated using the unit-cost edit distance, defined as the minimum number of insertions, deletions, and substitutions required to transform one sequence into another. The *edit similarity* of two sequences is defined as 1 − *d*/*L*, where *d* is their edit distance and *L* is the length of the longer sequence. As a fundamental metric for biological sequences, designing LSH functions for the edit similarity has received interest in theoretical computer science (Bar-Yossef et al. 2004) and become increasingly critical given the exponential growth of genomic data. However, unlike Hamming, Jaccard, and angular similarities, which have well-established LSH families, the progress on LSH families for edit similarity has been limited. The major challenge is that edit distance is not a normed metric, due to insertions and deletions that “misalign” the dimensions.

In this section, we describe two provable (Order Min Hash and SubseqHash) and one heuristic (Strobemer) LSH for edit distance. We note that these are primarily used as seeding or bucketing methods that tolerate errors (i.e., edits) in the data; as they are not similarity estimators, they are not usually used for estimating the edit distance directly.

### Order min hash

Order Min Hash (OMH) can be viewed as an extension of MinHash that accounts for order as well as makes use of multiple sampling (Marçais et al. 2019a). OMH first converts the input sequence *S* to a set of *n* pairs *M*(*S*) := {(*s*_1_, *l*_1_), …, (*s*_*n*_, *l*_*n*_)}, where *s*_*i*_ is the *i*th *k*-mer and *l*_*i*_ is its label. The label of *s*_*i*_ is the integer indicating the number of prior occurrences of *s*_*i*_ in *S*. For example, let *k* = 2 and *n* = 10; for input sequence *S* = GAAATTCAATC we have *M*(*S*) = {(GA, 0), (AA, 0), (AA, 1), (AT, 0), (TT, 0), (TC, 0), (CA, 0), (AA, 2), (AT, 1), (TC, 1)}.

Let π be an order over all possible (*k*-mer, label) pairs, that is, π is a permutation of Σ^*k*^ × [*n*]. The OMH hash function *h*_π_(*S*) picks the first ℓ pairs in *M*(*S*) according to π, for some predefined parameter ℓ. It then discards their labels and concatenates the *k*-mers following their original order of appearance in *S*. The value of *h*_π_(*S*) is this sequence of length ℓ*k*. Continuing the above example, suppose for the sake of simplicity that π ranks (*k*-mer, label) pairs in decreasing order of their labels, breaking ties in favor of lexicographically larger *k*-mers. For ℓ = 5, the first ℓ pairs in *M*(*S*) are then (AA, 2), (TC, 1), (AT, 1), (AA, 1), and (TT, 0). Concatenating them in the order they appear in *S* results in the hash value *h*_π_(*S*) = AATTAAATTC.

OMH is the family of the above hash functions, defined over all possible permutations π; formally, $H\;:=\{h_{\pi }|\pi \;\mathrm{is}\;a$ $\mathrm{permutation}\;\mathrm{of}\;\Sigma^{k}\times [n]\}$. OMH was proven by Marçais et al. (2019a) to be a LSH for the edit similarity: for any 2 ≤ ℓ ≤ *n* and any 1 > *s*_1_ ≥ *s*_2_ > 0, there exists *p*_1_ and *p*_2_ such that the family *H* is (*s*_1_, *s*_2_, *p*_1_, *p*_2_)-sensitive for the edit similarity.

OMH is mainly of theoretical interest, showing that it is possible to directly design LSH functions for the edit similarity without relying on embeddings. Marçais et al. (2019a) demonstrated on simulated data that sketches based on OMH are more sensitive to edits and outperforms MinHash sketches on phylogeny reconstruction. Nevertheless, tools such as Mash (Ondov et al. 2016) that “correct” the Jaccard similarity estimation by MinHash to an edit estimation using probabilistic models tend to perform better on benchmarking tests (Zielezinski et al. 2019).

### SubseqHash

SubseqHash (Li et al. 2023) follows the set-min framework but picks the smallest *subsequence* in an input sequence. Recall that unlike a substring, a subsequence selects potentially nonconsecutive positions from the input sequence. SubseqHash takes two parameters *m* and *k*, where *m* indicates the length of the input sequence and *k* indicates the length of the subsequence (i.e., length of the output hash value). Let *M*(*S*) be the set of all length-*k* subsequences of *S* and let π be a permutation over all possible length-*k* sequences. SubseqHash maps an input length-*m* sequence onto its smallest length-*k* subsequence according to π, formally written as

$$h_{\pi }(S)\;:=\arg\min_{z\in M(S)}\pi (z).$$

For example, let *m* = 5, *k* = 3, and *S* = ACGCA; the resulting length-*k* subsequence set is *M*(*S*) = {ACG, ACC, ACA, AGC, AGA, CGC, CGA, CCA, GCA}. If we assume for the sake of this example’s simplicity that π is the lexicographic order, then *h*_π_(*S*) = ACA.

Let $H\;:=\{h_{\pi }|\pi \;\text{is a permutation of}\;\Sigma^{k}\}$ be a family of hash functions over all possible permutations. When selecting π uniformly at random, every length-*k* subsequence has an equally likely chance of having the smallest rank. Therefore, similar to the set−min hash function, we have

$$\underset{h_{\pi }\in H}{\mathrm{Pr}}[h_{\pi }(S_{1})=h_{\pi }(S_{2})]=\frac{\left|M(S_{1})\cap M(S_{2})\right|}{\left|M(S_{1})\cup M(S_{2})\right|},$$

where the right term is the Jaccard similarity between two sets of subsequences. Therefore, *H* is a similarity estimator for the Jaccard similarity between the sets of constituent subsequences.

Even stronger, we can prove that *H* is an LSH for unit-cost edit similarity. The intuition behind is that two sequences admit a small unit-cost edit distance *if and only if* they share long subsequences (i.e., *k* is close to *m*). Consider two length-*m* sequences *S*_1_ and *S*_2_ with an edit distance between them *d*. If *d* is small enough (*d* ≤ *m* − *k*), then *S*_1_ and *S*_2_ are guaranteed to share a length-*k* subsequence. Therefore, $M(S_{1})\cap M(S_{2})\neq \emptyset$. A loose estimation gives $|M(S_{1})\cap M(S_{2})|\geq 1$ and $|M(S_{1})\cup M(S_{2})|\leq {\left|\Sigma \right|}^{k}$. Hence, for *p*_1_ : = 1/|Σ|^*k*^, $\mathrm{Pr}[h_{\pi }(S_{1})=h_{\pi }(S_{2})]\geq p_{1}$. If *d* is large enough (*d* ≥ 2(*m* − *k*) + 1), then, via a standard pigeonhole argument (Jones and Pevzner 2004), *S*_1_ and *S*_2_ are guaranteed to not share any length-*k* subsequence. Therefore, $M(S_{1})\cap M(S_{2})=\emptyset$, and, for *p*_2_ = 0, $\mathrm{Pr}[h_{\pi }(S_{1})=h_{\pi }(S_{2})]=p_{2}$. This argument shows that the family *H* is a (*s*_1_, *s*_2_, *p*_1_, *p*_2_)-sensitive LSH for the edit similarity, where *s*_1_ = 1 − (*m* − *k*)/*m* and *s*_2_ = 1 − (2(*m* − *k*) + 1)/*m*. Moreover, because *p*_2_ = 0, OR-amplification can be used to boost sensitivity while retaining perfect precision (Li et al. 2023).

However, the computation of *h*_π_(*S*) poses a challenge because the size of *M*(*S*) is exponential in the length of the subsequence. (As a comparison, for OMH, *M*(*S*) is linear.) A brute-force approach that computes π(*z*) for every *z* ∈ *M*(*S*) is therefore intractable. To overcome this, SubseqHash proposes an alternative, smaller, family of permutations, named the ABC order. The definition of this order is too involved for this survey, but the interested reader can refer to Li et al. (2023), which contains the formal definition (in Sec. 2.4) and an example (in Supplementary Note 3). If π is an ABC order, then *h*_π_(*S*) can be computed in polynomial time using dynamic programming.

SubseqHash is then defined as the hash family $H_{1}\;:=\{h_{\pi }|\pi \;\text{is an ABC order over}\;\Sigma^{k}\}$. Unlike *H*, *H*_1_ is not a similarity estimator for Jaccard of subsequences and hence is not a provable LSH for the edit similarity. However, Li et al. (2023) demonstrated using experiments that $\underset{h_{\pi }\in H_{1}}{\mathrm{Pr}}[h_{\pi }(S_{1})=h_{\pi }(S_{2})]$ approximates the Jaccard similarity for subsequences well and showed that *H*_1_ performs well as a heuristic LSH for unit-cost edit distance in practice.

SubseqHash outperforms *k*-mer based seeding methods on multiple sequence comparison applications, including read mapping, overlap detection, and sequence alignment (Li et al. 2023). It was shown that OR-amplification improves the practical accuracy of SubseqHash, albeit increasing the run time. A followup work SubseqHash2 (Li et al. 2025) employs an improved algorithm combined with SIMD acceleration to achieve nearly identical accuracy while substantially speeding up OR-amplified SubseqHash.

### Strobemers

Another LSH heuristic for edit similarity is the strobemer hash function (Sahlin 2021). Given parameters *l* and *t* ≥ 2, the idea is to extract *t* nonoverlapping substrings of length *l* (each of which is called a strobe) and concatenate them into a hash value called a strobemer. For example, for *S* = GTTCGTCGAATC and *l* = 2 and *t* = 3, the strobes might be given by the 2-mers starting at the red positions (i.e., GT, CG, and AA) and the strobermer would be GTCGAA. There are two more parameters, *w*_min_ and *w*_max_ (with *w*_min_ < *w*_max_), which define the *t* windows of starting positions from which the strobes are chosen. The first window is a special case and is just the first position, forcing the first strobe to be the first *l*-mer in *S*. The rest of the windows are of length *w*_max_ − *w*_min_ + 1, with the *i*th window starting at position 1 + (i − 2)*w*_max_ + *w*_min_. For example, with *w*_min_ = 3 and *w*_max_ = 5, the windows of starting positions are defined as [G]TT[CGT]CG[AAT]C. A strobemer is generated by choosing a single starting position from each window and concatenating the extracted strobes. We also require that $l\leq w_{\min}$ in order to guarantee that the strobes do not overlap. Finally, we note that the length of *S* should be exactly $l+(t-1)\cdot w_{\max}$, to fit all the windows; we refer to *S* as a super-window below. In applications, strobemers are often extracted from sliding super-windows of a long sequence.

We view strobemers as a heuristic LSH. Specifically, for two super-windows with a small edit distance, there is a high chance that the edits occur between the selected strobes, so that the resulting strobemer (i.e., the concatenation of strobes) remains unchanged and yields a hash-collision. In contrast, when two super-windows differ by many edits, it is unlikely that the edits will leave the extracted strobes unaffected, resulting in different strobes being chosen. Experiments show that strobemers are more robust to higher mutation rates than *k*-mers and spaced words.

Different variants of strobemers are formed by the different strategies used to select the strobe in each window. The minstrobe strategy selects the smallest *l*-mer from each window, where the minimization is defined with respect to a scattering hash function over all *l*-mers. For example, if we use the lexicographical order, then the strobes in our example are given by the starting positions in red: [G]TT[CGT]CG[AAT]C. The randstrobe strategy, on the other hand, selects the *l*-mer from the *i*th window such that the hash value of the concatenation of the previously selected (*i* − 1) strobes and this *i*th *l*-mer is minimized (again according to a scattering hash function). One metric that has been used to compare different versions of strobemers is *coverage*, defined as the fraction of all picked *l*-mers in the (long) sequence that appear as a strobe within a strobemer. High coverage is desirable for downstream applications. Experimental results show that the randstrobe strategy achieves higher coverage than the minstrobe strategy.

Strobemers and syncmers (Edgar 2021) were combined as a fast indexing method, leading to a new short-read aligner called Strobealign (Sahlin 2022). In order to allow better trade-offs between speed and sensitivity, Strobealign has an additional modification to allow strobes to be chosen from a pre-selected subset of the *l*-mer universe (in particular, it uses open syncmers of Edgar (2021)). This approach allows for faster computation (as the pool of candidate strobes are reduced in a window) while still maintaining tolerance to mutations and sequencing errors.

## Conclusion

Hash functions of various kinds are ubiquitous in nucleotide sequence analysis. Here, we attempted to survey existing approaches and to categorize them within a framework that highlights their properties while identifying their similarities and distinctions. We identified four categories, as follows. Scattering hash functions map sequences from a large universe into a smaller number of buckets in a way that tries to reduce the downstream harm of having collisions. Permutation hash functions scramble the order of sequences while not creating any collisions. Minimum perfect hash functions use prior knowledge about which subset of the universe will be hashed in order to avoid collisions on that subset. Locality-sensitive hash functions map sequences down to a smaller universe but control the collisions such that similar sequences are more likely to collide and dissimilar sequences are less likely to collide.

We do not cover cryptographic hash functions. The properties desirable in cryptographic applications include being hard to invert or being collision-resistant, meaning it is difficult to identify which pairs of elements collide. Such properties are not usually useful in nucleotide sequence analysis, where practical invertibility is actually useful for retrieving the original input easily.

We also do not cover quantization, which is the process of discretizing continuous data into buckets and using the bucket indices as hash values. Many sequencing technologies natively generate raw signal which is continuous. Tools such as RawHash (Firtina et al. 2023a, 2024) or Sigmoni (Shivakumar et al. 2024) take the signal (i.e., a real number) corresponding to a sequenced *k*-mer (e.g., *k* = 6) and hash it to a small integer. These hash functions can be viewed as LSHs, as they aim to map nearby values to the same bucket. This approach of working directly with the hashed raw signal has been applied to mapping (Shivakumar et al. 2024) and assembly (Firtina et al. 2026).

We have also omitted the weighted Jaccard similarity measure, which is similar to Jaccard but takes weights (e.g., the multiplicity of *k*-mers) into account. In its generality, the weights can be real numbers and there exists a similarity estimator called consistent weighted sampling (Ioffe 2010; Manasse et al. 2010). In the context of *k*-mer multi-sets where the multiplicity of a *k*-mer is used as its weight, OMH that extracts only one element (i.e., ℓ = 1) is a similarity estimator for the weighted Jaccard as well (Marçais et al. 2019a).

We do not explicitly discuss the RNA alphabet {A, C, G, U}, though most of everything we cover is equally applicable to RNA sequences. Protein sequences, however, require additional care due to their larger alphabet and the more complex relationships among amino acids; for example, unlike nucleotide sequences, it is unreasonable to assign uniform penalties to mismatches. Nonetheless, many hashing approaches have been successfully applied to amino acid sequences. For instance, Mash (Ondov et al. 2016) supports MinHash sketching for arbitrary alphabets, including amino acids. Domain-specific hashing algorithms and machine learning-based hashing methods have also been proposed for estimating protein sequence and structure similarity (Wong et al. 2023b; Han and Li 2024), but these approaches are beyond the scope of this survey.

One of the challenges in putting together this survey was an inconsistent use of terminology in the literature. First, there is widespread confusion about the difference between applying a nonrandom function to random data and applying a random hash function to nonrandom data. We have observed that experimental evaluations often fix a seed and test using randomly generated sequences (e.g., they measure regularity), as opposed to fixing adversarial data and testing using randomly generated seeds (e.g., measuring uniformity). To compound the issue, some papers use the term uniformity when regularity should be used instead. Second, there is a lack of clarity about the distinction between a hash function and a sketch, embedding, or a *k*-mer selection function. As mentioned earlier, we take the perspective that the outputs of a hash function are treated as ordered atomic values, while sketches or embeddings have a notion of distance between two different outputs. For example, two MinHash sketches are often compared using their Jaccard similarity (Broder 1997), which is more than can be done by simply checking if the two sketches are equal or not. There are also *k*-mer selection functions like syncmers (Edgar 2021), which take a *k*-mer and return a boolean value; we do not consider such functions as hash functions, even though they use randomization. In short, not all functions that use randomness are hash functions.

While surveying the field, it became apparent to us that many hash functions are developed and applied without being properly evaluated. For example, scattering hash functions are at best evaluated using the SMHasher benchmark; however, SMHasher does not in any way account for the spectrum-like property (Chikhi et al. 2021) of nucleotide sequence data. Another example are hash functions which are heuristically intended to be locality-sensitive. Although they are tested for their effect on downstream analysis, they often lack a more structured evaluation that targets the defining locality-sensitive properties. Overall, the field would benefit from genomic-sequence-specific benchmarks and, in general, more thorough forms of experimental evaluations.

## Competing interest statement

The authors declare no competing interests.

## Footnotes

5 This function is sometimes referred to as a minimizer hash function or as a MinHash with one sample hash function. This often depends on the application, so we therefore chose to introduce an application-neutral term here.

6 It is customary to use masks that start and end with a 1, although neither Califano and Rigoutsos (1993) nor Ma et al. (2002) imposed this requirement.
